# Supplementary material for: Rationale and design for studying organisation of care for intra-arterial thrombectomy in the Netherlands: simulation modelling study
Source: BMJ Open. 2020 Jan 7;10(1):e032754. doi: 10.1136/bmjopen-2019-032754 (PMC6955572; doi:10.1136/bmjopen-2019-032754)

Supplementary files

Table S1. Overview of data collection [Table S1.docx] – supplementary material for "Rationale and design of a simulation modelling approach for intra-arterial thrombectomy in the Netherlands" by Lahr M.M. et al.

Table 1. Overview of data collection**.**

**Item**	**Description**	**Source**
Prehospital items		
Symptom onset	Exact time of stroke onset, last seen well or symptoms noticed	CRF
Mode of referral	Initial call for help directed at either the GP, 911 emergency services or via self-transport	Ambulance database
Time first 911 call	Moment of first 911 call as documented at the control centre of the regional ambulance service	Ambulance database
Time second 911 call†	Moment of second 911 call as documented at the control centre of the regional ambulance service	Ambulance database
Time departure ambulance patient	Moment of ambulance departure in the direction of the patient	Ambulance database
Time departure ambulance hospital	Moment of ambulance departure in the direction of the PSC for transfer to the CSC	Ambulance database
Time ambulance arrival patient	Moment of ambulance arrival at the location of the patient	Ambulance database
Time ambulance arrival first hospital	Moment of ambulance arrival at the first hospital	Ambulance database
Time ambulance arrival second hospital†	Moment of ambulance arrival at the second hospital	Ambulance database
Time ambulance arrival PSC†	Moment of ambulance arrival at the PSC to transfer a patient to the CSC	Ambulance database
Procedures at location patient	All procedures performance by ambulance personnel at the location of the patient	Ambulance database
Postal code patient	Exact geographic location of the patient	Ambulance database
Postal code hospital	Exact geographical location of the destination hospital	Ambulance database
Intrahospital items		
Time of arrival ER first hospital	Moment of patient entry into the hospital electronic system	CRF
Time of arrival ER intervention hospital	Moment of patient entry into the hospital electronic system	CRF
Time of CT scan first hospital	Moment of CT scan performance in the first hospital	CRF
Time of CT scan second hospital	Moment of CT scan performance in the second hospital	CRF
Time of CTA scan first hospital	Moment of CTA scan performance in the first hospital	CRF
Time of CTA scan second hospital	Moment of CTA scan performance in the second hospital	CRF

Time of IV alteplase bolus (if given)	Moment of intravenous thrombolysis	CRF
Time of angiosuite arrival	Moment of patient arrival at the angiosuite	CRF
Time groin puncture	Moment of groin puncture	CRF
Time device attempt	Moment of device attempt	CRF
Time recanalisation	Time of recanalisation	CRF
Time of end procedure	Moment of sheath withdrawal/end of procedure	CRF

CRF indicates case report form; PSC, primary stroke centre; CSC, comprehensive stroke centre; CT, computed tomography; CTA, computed tomography angiogram.

†In case of intravenous thrombolysis at the primary stroke centre followed by intra-arterial thrombectomy at the comprehensive stroke centre (drip-and-ship approach).

Figure S1. Overview of IAT centres and ambulance services [Supplementary figure S1.tiff] - supplementary material for "Rationale and design of a simulation modelling approach for intra-arterial thrombectomy in the Netherlands" by Lahr M.M. et al.

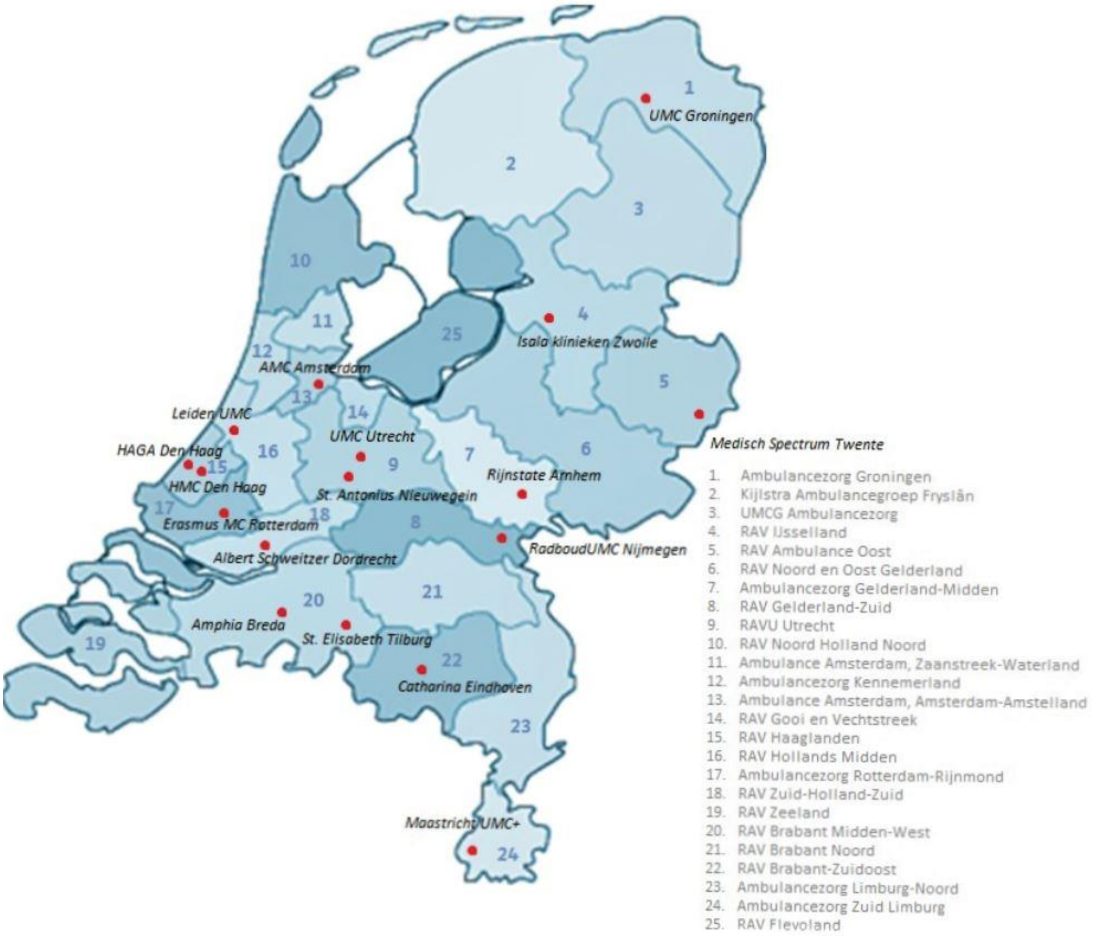

Supplement: Supplementary data [file bmjopen-2019-032754supp001.pdf]
