# Supplementary material for: Rationale and design for studying organisation of care for intra-arterial thrombectomy in the Netherlands: simulation modelling study
Source: BMJ Open. 2020 Jan 7;10(1):e032754. doi: 10.1136/bmjopen-2019-032754 (PMC6955572; doi:10.1136/bmjopen-2019-032754)

BMJ Open

BMJ Open is committed to open peer review. As part of this commitment we make the peer review history of every article we publish publicly available.

When an article is published we post the peer reviewers' comments and the authors' responses online. We also post the versions of the paper that were used during peer review. These are the versions that the peer review comments apply to.

The versions of the paper that follow are the versions that were submitted during the peer review process. They are not the versions of record or the final published versions. They should not be cited or distributed as the published version of this manuscript.

BMJ Open is an open access journal and the full, final, typeset and author-corrected version of record of the manuscript is available on our site with no access controls, subscription charges or pay-per-view fees (http://bmjopen.bmj.com).

If you have any questions on BMJ Open's open peer review process please email info.bmjopen@bmj.com

BMJ Open

Rationale and design for studying organisation of care for intra-arterial thrombectomy in the Netherlands: a simulation modelling study

Journal:	*BMJ Open*
Manuscript ID	bmjopen-2019-032754
Article Type:	Protocol
Date Submitted by the Author:	03-Jul-2019

Complete List of Authors:	Lahr, Maarten ; Health Technology Assessment, Department of Epidemiology, Health Technology Assessment, Department of Epidemiology<br>Maas, Willemijn; University of Groningen, University Medical Centre Groningen, Neurology, Epidemiology<br>van der Zee, Durk-Jouke; Rijksuniversiteit Groningen Faculteit Economie en Bedrijfskunde, Operations<br>Uyttenboogaart, Maarten; University of Groningen, University Medical Centre Groningen, Neurology<br>Buskens, Erik; University of Groningen, University Medical Center Groningen, Epidemiology; University of Groningen, Faculty of Economics and Business, Department of Operations

Keywords:	Stroke < NEUROLOGY, STROKE MEDICINE, EPIDEMIOLOGY, Organisation of health services < HEALTH SERVICES ADMINISTRATION & MANAGEMENT

SCHOLARONE™
Manuscripts

Rationale and design for studying organisation of care for intra-arterial thrombectomy in the Netherlands: a simulation modelling study

Maarten M.H. Lahr[1], Ph.D.; Willemijn J. Maas[1,2], M.Sc; Durk-Jouke van der Zee[3], Ph.D; Maarten Uyttenboogaart[2,4], M.D., Ph.D.; Erik Buskens[1,3] M.D., Ph.D, for the CONTRAST investigators.

[1] *Health Technology Assessment, Department of Epidemiology, University of Groningen, University Medical Centre Groningen, Groningen, The Netherlands*

[2] *Department of Neurology, University of Groningen, University Medical Centre Groningen, Groningen, The Netherlands*

[3] *Department of Operations, Faculty of Economics & Business, University of Groningen, Groningen, The Netherlands*

[4] *Department of Radiology, University of Groningen, University Medical Centre Groningen, Groningen, The Netherlands*

Co-authors addresses:
Willemijn Maas w.j.maas@umcg.nl
Durk-Jouke van der Zee d.j.van.der.zee@rug.nl
Maarten Uyttenboogaart m.uyttenboogaart@umcg.nl
Gert-Jan Luijckx g.j.luickx@umcg.nl
Erik Buskens e.buskens@umcg.nl

Key words: stroke, intra-arterial thrombectomy, intravenous thrombolysis, simulation modelling, organisational model, probability.

Word count all sections: 3951
Word count main text: 2875
Number of tables: 0
Number of figures: 2
Supplementary tables: 1
Supplementary figures: 1

Corresponding Author:
Maarten M.H. Lahr
University Medical Centre Groningen, Department of Epidemiology
P.O. Box 30001. 9700 RB Groningen, The Netherlands
Telephone: + 31 50 361 43 86
E-mail: m.m.h.lahr@umcg.nl

For peer review only

Abstract

Introduction – The introduction of intra-arterial thrombectomy (IAT) challenges acute stroke care organisations to provide fast access to acute stroke therapies. Parameters of pathway performance include distances to primary and comprehensive stroke centres, time to treatment and availability of ambulance services. Further expansion of IAT centres may increase treatment rates, yet, could affect efficient use of resources and quality of care due to lower treatment volume. The aim was to study the organisation of care and patient logistics of IAT for ischaemic stroke patients in the Netherlands.

Methods and analyses – Using a simulation modelling approach, performance of sixteen primary and comprehensive stroke centres offering IAT in the Netherlands will be quantified. Patient data concerning both pre- and intrahospital pathway logistics will be collected and used as input for model validation. A previously validated simulation model for intravenous thrombolysis (IVT) patients will be expanded with data of the MR CLEAN Registry and trials performed in the CONTRAST consortium to represent patient logistics, time delays and outcomes in IAT patients. Simulation experiments aim to assess effectiveness and efficiency of alternative network topologies, i.e. IAT with or without IVT at the nearest primary stroke centre versus centralised care at comprehensive stroke centre (CSC). Primary outcomes are IAT treatment rates and patient disability according to the modified Rankin Scale. Secondary outcomes include onset-to-treatment time and resource use. Mann-Whitney U and Fisher's exact tests will be used to estimate differences for continuous and categorical variables. Model and parameter uncertainty will be tested using sensitivity analyses.

Ethics and dissemination – This will be the first study to examine the organisation of acute stroke care for IAT delivery on a national scale using discrete event simulation. There are no ethics or safety concerns regarding the dissemination of information, which includes publication in peer-reviewed journals and (inter)national conference presentations.

For peer review only

Strengths and limitations of the study

- The proposed simulation modelling study collects patient level data from all interventions centers in the Netherlands that provide IAT for acute ischaemic stroke patients.
- Information from prehospital stroke services including emergency medical services are included in the model.
- Information on costs associated with pathway set-up and innovations foreseen in acute stroke treatment are included.
- Model results are estimations which have to be tested in clinical practice.
- Input parameters for model building contain estimations of time delays and diagnostic procedures that may have changed over time.

For peer review only

INTRODUCTION

For acute ischaemic stroke patients, reperfusion therapies comprise intravenous thrombolysis (IVT) administered up to 4.5 hours after onset, and intra-arterial thrombectomy (IAT) up to 6 hours and in selected patients even up to 24 hours.[1-7] Functional recovery following treatment is strongly determined by the time interval between symptom onset and reperfusion. For every 9-minute delay along the care pathway, 1 in every 100 patients treated with IAT has a worse disability outcome (higher modified Rankin Scale score by 1 or more levels).[8] As such, timely arrival at the hospital and minimising the overall onset-to-treatment time is of critical importance. Following symptom onset patients are typically transported to the nearest hospital capable of administering IVT, which can be either a Primary Stroke Centre (PSC) or a Comprehensive Stroke Centre (CSC). In parallel with administering the bolus for IVT additional neuroimaging is performed in order to detect large vessel occlusion (LVO). In case of a LVO patients may become eligible for IAT after which transfer to a CSC is arranged, if patients are initially admitted to a PSC.

The transfer of patients to a CSC following IVT diagnostic work-up and/or treatment at a PSC is called the drip-and-ship (DS) approach. In addition to the DS approach patients may be transported directly to the CSC bypassing the PSC, which is called the Mothership (MS) approach. Currently there are no formal policies supporting the latter, as it necessitates accurate prehospital triage objectifying LVO with sufficient accuracy. Timely patient arrival and treatment with reperfusion therapies depends on a number of factors such as the geographical distribution and distances between PSCs and CSCs in relation to the location of the patient. Arrangements made by local emergency medical services concerning referral patterns of suspected stroke victims, and the quality and set-up of road networks also play a role, as does potential traffic congestion.

In the Netherlands sixteen medical centres that participated in the MR CLEAN trial are currently reimbursed for IAT. However, these hospitals are unevenly distributed leaving certain regions potentially underserved while in some areas there is a surplus of IAT centres. Insight into factors determining the potential optimal and most efficient way to organise acute stroke care is currently lacking but urgently needed to support clinicians and decision-makers. A key question is: what would be the optimal distribution of CSCs within a region, taking into account the effects of additional travel distances, available clinical expertise, treatment volumes and capacity of hospitals? Are there regions under- or overserved with CSCs and what would be the potential consequences of adding/removing CSCs within specific regions?

In this study, we aim to quantify pathway logistics, i.e. stroke onset and time delays associated with pre-hospital and intra-hospital care services among patients treated with IAT with or without IVT. From this baseline assessment a simulation model will be built that describes all activities and treatments performed along the acute stroke pathway. A generic model will be developed based on the set-up of IAT delivery in the region of Northern Netherlands, and extended using results of the MR CLEAN registry[9] and data of 4 randomised clinical trials (RCTs) performed in the 'Collaboration for new treatments in acute stroke (CONTRAST)' consortium (www.contrast-consortium.nl). Simulation experiments will be performed to assess effectiveness and efficiency of alternative network topologies, i.e. primary and comprehensive stroke services (DS approach) versus a service delivery based on comprehensive and centralised care (MS approach). Model parameters will be extended by assigning unit costs to activities allowing for economic analyses.

For peer review only - http://bmjopen.bmj.com/site/about/guidelines.xhtml

Aims and hypotheses

Primary aim: to develop a simulation model for IAT in the Netherlands and to extend the model to reflect regional differences.

Secondary aims: to estimate the effectiveness of alternative network topologies on IVT and/or IAT treatment rates, geographical access to CSCs, time to treatment, treatment volumes and patient disability as measured by the modified Rankin Scale (Mrs). Also, potential areas for pathway improvements in regional stroke care systems will be identified.

Tertiary aims: to extend the model with costs estimations of procedures and activities performed along the acute stroke pathway allowing for elaborate economic evaluation. Also, the assessment and potential implementation of new technologies such as the mobile stroke unit is foreseen through modelling.

METHODS

Study design

This study uses discrete event simulation modelling in which care pathways can be represented 'in silico' by quantifying pathway performance.[10-12] In case of acute stroke treatment, information on time delays sustained by patients and diagnostic steps performed up to treatment with IVT and IAT are collected. Previous research has demonstrated that simulation models for representing the acute stroke pathway can be truthfully developed in different settings.[13-17] Typically, simulation modelling involves a number of consecutive steps including model building, validation and experimentation. Model building starts with the conceptual representation of care pathways in building block such as incidents, time delays, queues, resources and ultimately outcome. Next the model needs to be populated through collection of empirical data reflecting actual pathway performance, for which a combination of real-world datasets are used. For hospital items we will rely on case report forms (CRFs) collected in the RCTs in the CONTRAST consortium involving sixteen hospitals in the Netherlands that provide IAT. In addition, collaboration with regional ambulance services will be set up in order to collect prehospital items such as time of 911 call, time spent on scene, transportation time to the hospital and general referral patterns. Real-world data collected in each step of the pathway will be analysed to distinguish statistical distributions using the statistical software package ExpertFit.[18] Based on the statistical distributions identified hypothetical patients passing through the model will be assigned a certain time delay, and diagnostic accuracy for each step along the pathway. Model outcomes include the proportion of patients treated with IAT, time to treatment and functional outcome after 90 days. These results will then be validated comparing them to observed time delays and outcomes observed in clinical practice. Following model validation, experiments will be performed in which scenarios of alternative set-ups of the organisational models will be evaluated. Descriptions of the acute stroke pathway in both the DS and MS organisational models in Northern Netherlands are presented in Figures 1 and 2.

For peer review only

Figure 1. Description of the drip-and-ship model.

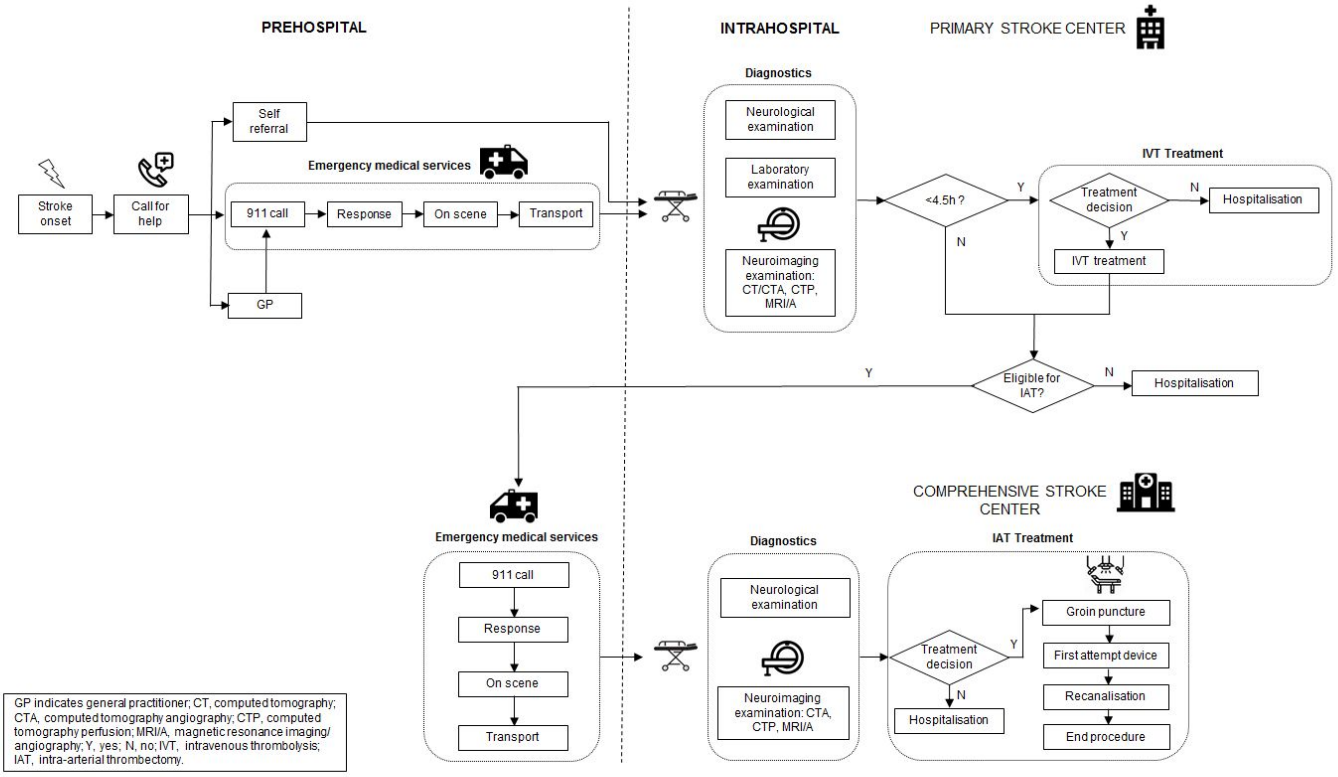

Figure 2. Description of the Mothership model.

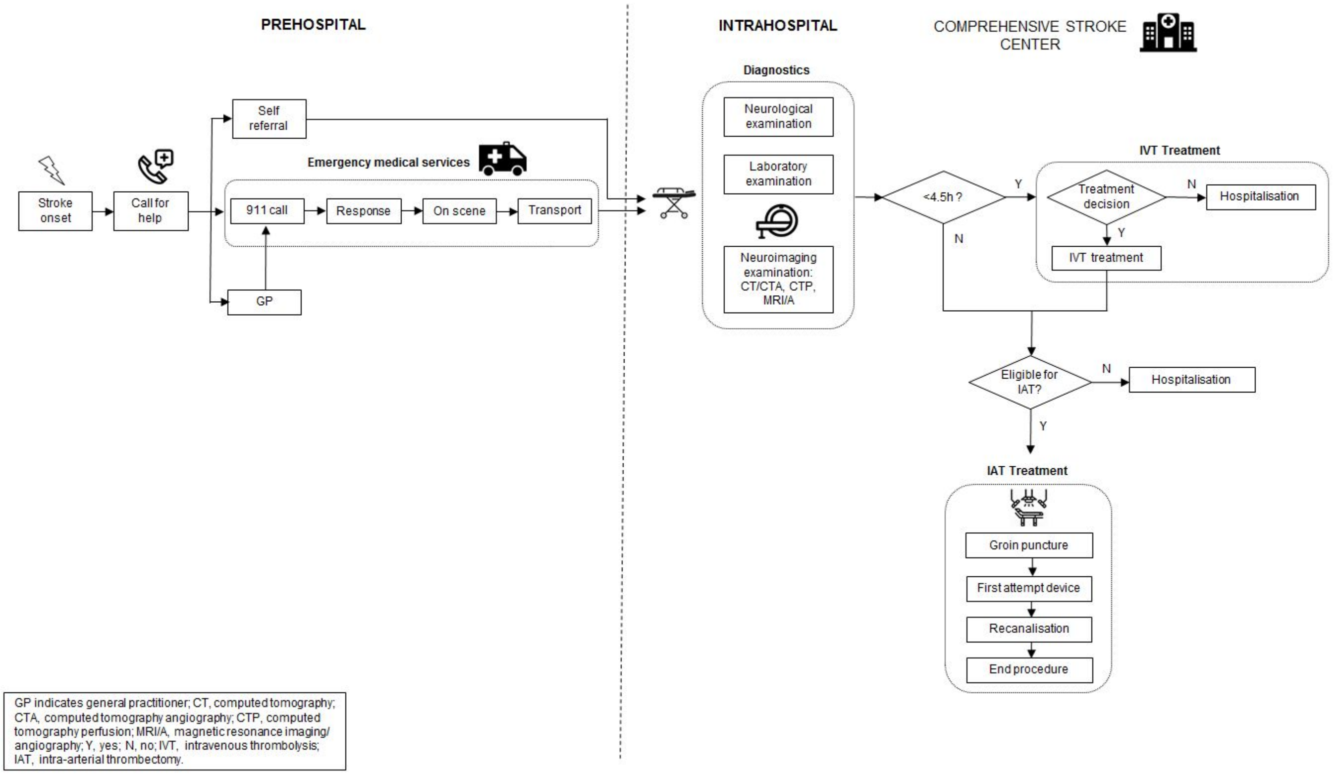

Study population

For this study we will use data from acute stroke patients that received IAT with or without IVT. A baseline model will be developed using a subset of patient information collected in the ongoing cohort study MR CLEAN Registry. Following the initial model patients treated in other regions in the Netherlands will be included. In later stages of model development alternative treatment modalities may become apparent such as treating patient with IAT up to 24 hours or including patients that receive prehospital treatment in a mobile stroke unit. In these cases the parameters underlying the model will be changed accordingly.

Public and patient involvement

Patients and public were involved in the conception of the topics to be addressed in the CONTRAST consortium. Study results will be disseminated through newsletters, poster presentations, and publications in newspapers, lay-men journals, and publication in peer-reviewed journals.

Data to be collected

An overview of all data items collected for the simulation model is presented in a table as supplementary material (Table S1. Overview of data collection).

Hospital data

Within CONTRAST 4 RCTs are performed that serve as input for the proposed simulation modelling studies: MR ASAP (NL60258.078.17), MR CLEAN LATE (NL58246.078.17), MR CLEAN MED (ISCRTN76741621), MR CLEAN NO IV (ISRCTN80619088). Each intervention centre uses a standardised, customised and web-based electronic CRF to document all steps in the care process. All data will be entered locally in web-based database (OpenClinica). A copy of the CRF documents used in the clinical trials can be found online (www.contrast-consortium.nl). All studies aim to collect a large set of items containing, among others, a description of the workflow including time of symptom onset or last seen well, time of arrival of the first hospital and whether the patient was transferred from another hospital. Within the hospital, time to computed tomography (CT) scan and advanced imaging (computed tomography angiography/ magnetic resonance angiography/digital subtraction angiography/perfusion imaging) are collected according to local procedures. Also start of IVT (bolus infusion), patient arrival in the angiosuite, groin puncture, device attempts, recanalisation and sheath withdrawal/end of procedure are collected. In addition anesthetic team presence and management procedures are recorded.

Prehospital data

For the collection of prehospital data collaboration with regional ambulance services is being set-up. Items will include time delays, diagnostics and geographic information on referral patterns. Time delays include the time of 911 call, arrival at the location of the patient, departure at the location of the patient and arrival at the hospital. Diagnostic items include mode of referral (general practitioner, 911 or other), initial working diagnosis set by 911 dispatch and ambulance personnel and the level of urgent transportation (i.e. blue lights or

For peer review only

other). Geographical information includes the postal codes of the location of the patient, departure location of the ambulance vehicle and location of the PSC and/or CSC.

Baseline data

Prior to the CONTRAST study, all hospitals contributed to the MR CLEAN Registry, an online and ongoing database to monitor implementation and safety of IAT in the Netherlands.[9] For CONTRAST the variables are similar to those collected in the MR CLEAN Registry. In addition, prehospital information available at regional ambulance services will be collected.

The baseline discrete event simulation model will be developed using data from 296 patients that received IAT at the University Medical Centre Groningen between July 2014 and November 2017. For these patients intrahospital time delays and diagnostic steps were already collected as part of the ongoing MR CLEAN Registry and subsequently used for model development and validation. In addition, prehospital items as described above were collected in collaboration with regional ambulance services.

Outcome measures

Outcomes will be estimated by the simulation model using parameter distributions as observed in clinical practice using real-world data.

Primary outcomes include IAT treatment rates and patient disability according to the modified Rankin Scale (Mrs).

Secondary outcomes include process times such as symptom onset to hospital arrival, door to needle and groin puncture, arrival angiosuite, recanalization, onset-to-treatment time of IVT and/or IAT and interhospital transportation times for those patients that were transferred to a CSC after initial diagnosis and/or treatment with IVT at a PSC.

Tertiary outcomes include treatment volume, direct costs, indirect costs and societal costs.

Data management and ethics

In order to link patient data collected after study enrollment in the hospital to corresponding data collected by ambulance services, identifiable information from patients will be used under a strict protocol compliant with the Dutch Personal Data Protection Act. This protocol meets the criteria for a waiver of consent from the University Medical Centre Groningen Institutional Review Board. Prior to the exchange of information, a data transfer and processing agreement was agreed upon and signed by each provider of regional ambulance services.

Study sites

Sixteen hospitals providing IAT will participate. In addition, regional ambulance services in the catchment area of intervention centres will be asked to participate. The overview of intervention centres, their catchment area and regional ambulance services active in the region are provided as supplementary material (Figure S1. Overview of IAT centres and ambulance services).

For peer review only

Statistical analyses

Descriptive statistics will be used to characterise the population and regional differences. Mann-Whitney U and Fisher's exact tests are used to estimate the differences for continuous and categorical variables. Model and parameter uncertainty will be tested using sensitivity analyses.

Approach to missing data

Multiple imputation techniques will be used to handle missing data. The impact of missing data will be analysed by performing sensitivity analyses. Once missing data or errors are identified, all corrections made will be documented.

Model validation

Internal validation will be performed by comparing model performance with observed performance of the stroke system to ascertain whether the model represented the real system accurately. External validation of the baseline model will be performed by using the pathway performance of other PSCs and CSCs participating in the CONTRAST consortium. As such, the model developed for one region will be extended to other parts of the Netherlands by changing the distributions underlying the simulation model. This means that the model is re-populated by real-world data originating from different regions, including both intrahospital and prehospital data. As we will adopt one uniform method for data collected in both the pre- and intrahospital pathway, similar procedures for model building, validation and experimentation can be followed.

Study organisation

The CONTRAST consortium is a nationwide collaboration of clinical and translational scientists from all academic and large clinical centres who want to act together to improve the treatment of acute stroke in the Netherlands. Four large acute stroke trials to test novel treatment strategies will be performed including: prehospital augmentation of collateral blood flow and blood pressure reduction (MR ASAP), antithrombotics to prevent microvascular occlusion after IAT (MR CLEAN MED), immediate IAT without preceding thrombolysis (MR CLEAN NO IV) and IAT in the 6 to 24 hour time window (MR CLEAN LATE). Discrete event simulation modelling is applied with data from these trials to optimise acute stroke care delivery.

DISCUSSION

This simulation modelling study will evaluate the set-up of acute stroke services for patients receiving IAT in a specific geographical setting, i.e., the Netherlands. However, the model foreseen may also serve as a planning and evaluation tool for other regions and countries. Already from an early phase onwards the potential consequences of the clinical trials performed in CONTRAST on the organisation of acute stroke care will be assessed. Benefits of innovations foreseen in prehospital as well as hospital based diagnosis and treatment will be estimated on a regional and national scale. Likewise, the optimal organisation ensuing from these re-organising care processes may be identified and taken into account given

For peer review only

available resources and capacity. Experiments from simulation models will help to elucidate factors determining optimal stroke pathway set-up and identify potential targets for further optimisation of pathway performance. Simulation modelling also allows for making exact estimations on effects on patients health and costs when adding or removing IAT centres in specific regions, thereby supporting clinicians and policy makers in decision making.[15]

Our simulation modelling approach has several strengths. First, the prospective design and uniform methods for data collection both in the pre- and intrahospital phase will ensure a high quality of data capturing. The use of real-world data will support the external validation of results obtained and is a unique feature of the proposed modelling study. Also in terms of efficiency simulation modelling is an attractive option, as it utilizes already collected information in clinical trials and by ambulance services. Second, the model will have a comprehensive scope, contrary to current improvement approaches characterised by mono-dimensional interventions.[19-21] Proposed solutions following such an approach are per definition finite, and cannot not solve implementation problems such as how many CSCs would a region need or where they should ideally be located. Data used for model input will be collected in various regions of the Netherlands reflecting differences in access to PSCs and CSCs, road networks and potential traffic congestion. Third, the model builds on a previously developed simulation model for IVT treatment in Northern Netherlands.[10] As such we have demonstrated that model building and validation is feasible. Finally, the model can be extended to include unit costs for elaborate economic analyses. The comprehensive character of the modelling approach also allows considering potential interaction effects between variables, and has a quadruple dimensional approach in terms of measuring outcomes, as it can capture effects on clinical outcomes, time to treatment, referral patterns and costs of processes associated with IVT and IAT delivery.

Serving as a precursor for clinical validation, results obtained from modelling studies should always be interpreted with care as these results do not represent actual patients. However, from an economics and business approach each treatment can be represented as the sum of time delays and diagnostics performed along the care pathway, and as such can be quantified and in turn represented 'in silico' by computer models. One of the main questions is whether there is benefit in bypassing the nearest PSC to transport the patient directly to a CSC. At the moment there is no consensus which prehospital scale to use in order to quantify stroke severity, and no agreed upon triage instrument exists to guide transfer of patients to specific hospitals. Typically, in case of a positive trial results in CONTRAST this will have a direct impact on the organisational model for IAT delivery, either by suggesting to bypass PSCs (MR CLEAN NO IV), by extending the time window for reperfusion therapies (MR CLEAN LATE) or to change prehospital stroke management (MR ASAP). As a consequence alternative set-up of services is foreseen, in which accurate estimations on effectiveness, time to treatment and cost-effectiveness are required to support decision-making.

CONCLUSION

The proposed modelling study will investigate current organisational models for IAT delivery in the Netherlands. Based on input from real-world clinical studies performed in the CONTRAST consortium insight into current treatment delays and workflow performance of IAT centres will be obtained. Next, simulation modelling studies will support design of optimal distribution of IAT centres, in order to improve patient outcomes and increase the proportion of patients eligible for IAT.

For peer review only - http://bmjopen.bmj.com/site/about/guidelines.xhtml

Acknowledgements We acknowledge the support of the Netherlands Cardiovascular Research Initiative which is supported by the Dutch Heart Foundation (CVON2015-01: CONTRAST), the support of the Brain Foundation Netherlands (HA2015.01.06), and the support of Health~Holland, Top Sector Life Sciences & Health (LSHM17016) and of Medtronic.

Contributors ML, WM, DJZ, MU and EB designed the study with MU and EB as principal investigators. ML, MU and EB applied for, received and organised study funding. ML drafted the manuscript, WM, DJZ, MU and EB critically revised the manuscript for intellectual content and approved the final version of the manuscript for publication.

Funding The CONTRAST consortium is supported by Netherlands Cardiovascular Research Initiative, an initiative of the Dutch Heart Foundation, by the Brain Foundation Netherlands and powered by Health~Holland, Top Sector Life Sciences and receives unrestricted funding from Medtronic.

Competing interest statement The authors declare that there is no conflict of interest.

References

1 . Tissue plasminogen activator for acute ischemic stroke. The National Institute of Neurological Disorders and Stroke rt-PA Stroke Study Group. *N Engl J Med* 1995;333:1581-7.

2 Berkhemer OA, Fransen PS, Beumer D, et al. A randomized trial of intraarterial treatment for acute ischemic stroke. *N Engl J Med* 2015;372:11-20.

3 Campbell BC, Mitchell PJ, Kleinig TJ, et al. Endovascular therapy for ischemic stroke with perfusion-imaging selection. *N Engl J Med* 2015;372:1009-18.

4 Goyal M, Demchuk AM, Menon BK, et al. Randomized assessment of rapid endovascular treatment of ischemic stroke. *N Engl J Med* 2015;372:1019-30.

5 Jovin TG, Chamorro A, Cobo E, et al. Thrombectomy within 8 hours after symptom onset in ischemic stroke. *N Engl J Med* 2015;372:2296-306.

6 Saver JL, Goyal M, Bonafe A, et al. Stent-retriever thrombectomy after intravenous t-PA vs. t-PA alone in stroke. *N Engl J Med* 2015;372:2285-95.

7 Nogueira RG, Jadhav AP, Haussen DC, et al. Thrombectomy 6 to 24 Hours after Stroke with a Mismatch between Deficit and Infarct. *N Engl J Med* 2018;378:11-21.

8 Saver JL, Goyal M, van der Lugt A, et al. Time to Treatment With Endovascular Thrombectomy and Outcomes From Ischemic Stroke: A Meta-analysis. *JAMA* 2016;316:1279-88.

9 Jansen IGH, Mulder MJHL, Goldhoorn RB, et al. Endovascular treatment for acute ischaemic stroke in routine clinical practice: prospective, observational cohort study (MR CLEAN Registry). *BMJ* 2018;360:k949.

For peer review only

10 Lahr MM, van der Zee DJ, Vroomen PC, et al. Thrombolysis in acute ischemic stroke: a simulation study to improve pre- and in-hospital delays in community hospitals. *PLoS One* 2013;8:e79049.

11 Ramwadhdoebe S, Van Merode GG, Boere-Boonekamp MM, et al. Implementation by simulation; strategies for ultrasound screening for hip dysplasia in the Netherlands. *BMC Health Serv Res* 2010;10:75.

12 Heeg BM, Damen J, Buskens E, et al. Modelling approaches: the case of schizophrenia. *Pharmacoeconomics* 2008;26:633-48 doi:2682 [pii].

13 Holodinsky JK, Williamson TS, Demchuk AM, et al. Modeling Stroke Patient Transport for All Patients With Suspected Large-Vessel Occlusion. *JAMA Neurol* 2018;75:1477-86.

14 Phan TG, Beare R, Chen J, et al. Googling Service Boundaries for Endovascular Clot Retrieval Hub Hospitals in a Metropolitan Setting: Proof-of-Concept Study. *Stroke* 2017;48:1353-61.

15 Lahr MM, van der Zee DJ, Luijckx GJ, et al. Centralising and optimising decentralised stroke care systems: a simulation study on short-term costs and effects. *BMC Med Res Methodol* 2017;17:5,016-0275-3.

16 Monks T, Pitt M, Stein K, et al. Maximizing the Population Benefit From Thrombolysis in Acute Ischemic Stroke: A Modeling Study of In-Hospital Delays. *Stroke* 2012;43(10):2706-11.

17 Churilov L, Fridriksdottir A, Keshtkaran M, et al. Decision support in pre-hospital stroke care operations: a case of simulation to improve eligibility of acute stroke patients for thrombolysis treatment. Computer & Operations Research 2013; 40: 2208–218.

18 Law AM. ExpertFit Version 8 User's Guide. Tuscon, Arizona: Averill M. Law & Associates 2011.

19 Jeon SB, Ryoo SM, Lee DH, et al. Multidisciplinary Approach to Decrease In-Hospital Delay for Stroke Thrombolysis. *J Stroke* 2017;19:196-204.

20 Psychogios MN, Behme D, Schregel K, et al. One-Stop Management of Acute Stroke Patients: Minimizing Door-to-Reperfusion Times. *Stroke* 2017;48:3152-5.

21 Schregel K, Behme D, Tsogkas I, et al. Effects of Workflow Optimization in Endovascularly Treated Stroke Patients - A Pre-Post Effectiveness Study. *PLoS One* 2016;11:e0169192.

For peer review only

Supplementary files

Table S1. Overview of data collection [Table S1.docx] – supplementary material for "Rationale and design of a simulation modelling approach for intra-arterial thrombectomy in the Netherlands" by Lahr M.M. et al.

Table 1. Overview of data collection**.**

**Item**	**Description**	**Source**
Prehospital items		
Symptom onset	Exact time of stroke onset, last seen well or symptoms noticed	CRF
Mode of referral	Initial call for help directed at either the GP, 911 emergency services or via self-transport	Ambulance database
Time first 911 call	Moment of first 911 call as documented at the control centre of the regional ambulance service	Ambulance database
Time second 911 call†	Moment of second 911 call as documented at the control centre of the regional ambulance service	Ambulance database
Time departure ambulance patient	Moment of ambulance departure in the direction of the patient	Ambulance database
Time departure ambulance hospital	Moment of ambulance departure in the direction of the PSC for transfer to the CSC	Ambulance database
Time ambulance arrival patient	Moment of ambulance arrival at the location of the patient	Ambulance database
Time ambulance arrival first hospital	Moment of ambulance arrival at the first hospital	Ambulance database
Time ambulance arrival second hospital†	Moment of ambulance arrival at the second hospital	Ambulance database
Time ambulance arrival PSC†	Moment of ambulance arrival at the PSC to transfer a patient to the CSC	Ambulance database
Procedures at location patient	All procedures performance by ambulance personnel at the location of the patient	Ambulance database
Postal code patient	Exact geographic location of the patient	Ambulance database
Postal code hospital	Exact geographical location of the destination hospital	Ambulance database
Intrahospital items		
Time of arrival ER first hospital	Moment of patient entry into the hospital electronic system	CRF
Time of arrival ER intervention hospital	Moment of patient entry into the hospital electronic system	CRF
Time of CT scan first hospital	Moment of CT scan performance in the first hospital	CRF
Time of CT scan second hospital	Moment of CT scan performance in the second hospital	CRF
Time of CTA scan first hospital	Moment of CTA scan performance in the first hospital	CRF
Time of CTA scan second hospital	Moment of CTA scan performance in the second hospital	CRF

For peer review only

Time of IV alteplase bolus (if given)	Moment of intravenous thrombolysis	CRF
Time of angiosuite arrival	Moment of patient arrival at the angiosuite	CRF
Time groin puncture	Moment of groin puncture	CRF
Time device attempt	Moment of device attempt	CRF
Time recanalisation	Time of recanalisation	CRF
Time of end procedure	Moment of sheath withdrawal/end of procedure	CRF

CRF indicates case report form; PSC, primary stroke centre; CSC, comprehensive stroke centre; CT, computed tomography; CTA, computed tomography angiogram.

†In case of intravenous thrombolysis at the primary stroke centre followed by intra-arterial thrombectomy at the comprehensive stroke centre (drip-and-ship approach).

Figure S1. Overview of IAT centres and ambulance services [Supplementary figure S1.tiff] - supplementary material for "Rationale and design of a simulation modelling approach for intra-arterial thrombectomy in the Netherlands" by Lahr M.M. et al.

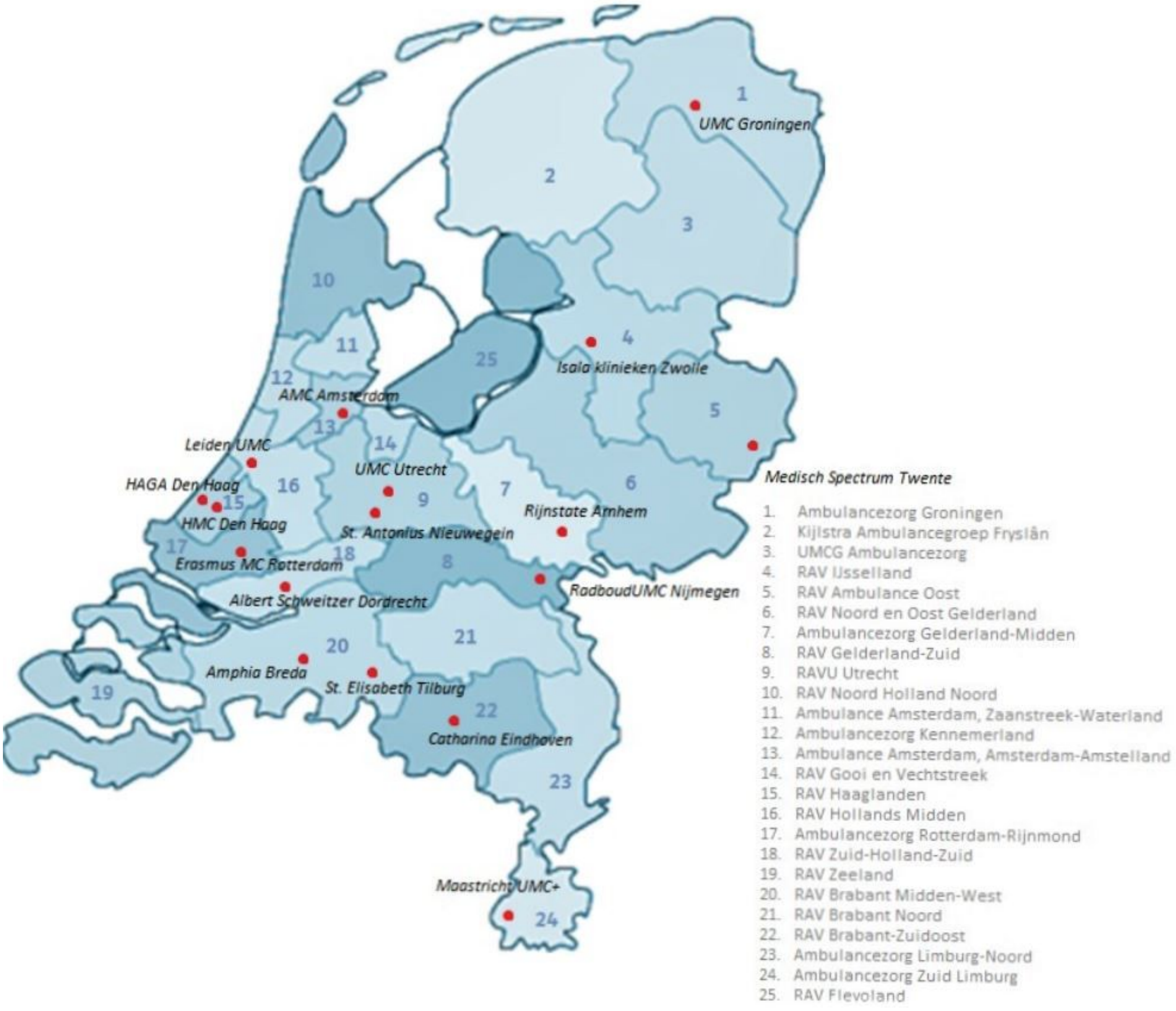

BMJ Open

Rationale and design for studying organisation of care for intra-arterial thrombectomy in the Netherlands: a simulation modelling study

Journal:	*BMJ Open*
Manuscript ID	bmjopen-2019-032754.R1
Article Type:	Protocol
Date Submitted by the Author:	31-Oct-2019
Complete List of Authors:	Lahr, Maarten ; Health Technology Assessment, Department of Epidemiology, Health Technology Assessment, Department of Epidemiology<br>Maas, Willemijn; University of Groningen, University Medical Centre Groningen, Neurology, Epidemiology<br>van der Zee, Durk-Jouke; Rijksuniversiteit Groningen Faculteit Economie en Bedrijfskunde, Operations<br>Uyttenboogaart, Maarten; University of Groningen, University Medical Centre Groningen, Neurology<br>Buskens, Erik; University of Groningen, University Medical Center Groningen, Epidemiology; University of Groningen, Faculty of Economics and Business, Department of Operations
**Primary Subject Heading**:	Neurology
Secondary Subject Heading:	Epidemiology, Health services research
Keywords:	Stroke < NEUROLOGY, STROKE MEDICINE, EPIDEMIOLOGY, Organisation of health services < HEALTH SERVICES ADMINISTRATION & MANAGEMENT

SCHOLARONE™
Manuscripts

Rationale and design for studying organisation of care for intra-arterial thrombectomy in the Netherlands: a simulation modelling study

Maarten M.H. Lahr[1], Ph.D.; Willemijn J. Maas[1,2], M.Sc; Durk-Jouke van der Zee[3], Ph.D; Maarten Uyttenboogaart[2,4], M.D., Ph.D.; Erik Buskens[1,3] M.D., Ph.D, for the CONTRAST investigators.

[1] *Health Technology Assessment, Department of Epidemiology, University of Groningen, University Medical Centre Groningen, Groningen, The Netherlands*

[2] *Department of Neurology, University of Groningen, University Medical Centre Groningen, Groningen, The Netherlands*

[3] *Department of Operations, Faculty of Economics & Business, University of Groningen, Groningen, The Netherlands*

[4] *Department of Radiology, University of Groningen, University Medical Centre Groningen, Groningen, The Netherlands*

Co-authors addresses:
Willemijn Maas w.j.maas@umcg.nl
Durk-Jouke van der Zee d.j.van.der.zee@rug.nl
Maarten Uyttenboogaart m.uyttenboogaart@umcg.nl
Erik Buskens e.buskens@umcg.nl

Key words: stroke, intra-arterial thrombectomy, intravenous thrombolysis, simulation modelling, organisational model, probability.

Word count all sections: 3951
Word count main text: 2875
Number of tables: 0
Number of figures: 2
Supplementary tables: 1
Supplementary figures: 1

Corresponding Author:
Maarten M.H. Lahr
University Medical Centre Groningen, Department of Epidemiology
P.O. Box 30001. 9700 RB Groningen, The Netherlands
Telephone: + 31 50 361 43 86
E-mail: m.m.h.lahr@umcg.nl

For peer review only

Abstract

Introduction – The introduction of intra-arterial thrombectomy (IAT) challenges acute stroke care organisations to provide fast access to acute stroke therapies. Parameters of pathway performance include distances to primary and comprehensive stroke centres, time to treatment and availability of ambulance services. Further expansion of IAT centres may increase treatment rates, yet, could affect efficient use of resources and quality of care due to lower treatment volume. The aim was to study the organisation of care and patient logistics of IAT for ischaemic stroke patients in the Netherlands.

Methods and analyses – Using a simulation modelling approach, performance of sixteen primary and comprehensive stroke centres offering IAT in the Netherlands will be quantified. Patient data concerning both pre- and intrahospital pathway logistics will be collected and used as input for model validation. A previously validated simulation model for intravenous thrombolysis (IVT) patients will be expanded with data of the MR CLEAN Registry and trials performed in the CONTRAST consortium to represent patient logistics, time delays and outcomes in IAT patients. Simulation experiments aim to assess effectiveness and efficiency of alternative network topologies, i.e. IAT with or without IVT at the nearest primary stroke centre versus centralised care at comprehensive stroke centre (CSC). Primary outcomes are IAT treatment rates and clinical outcome according to the modified Rankin Scale. Secondary outcomes include onset-to-treatment time and resource use. Mann-Whitney U and Fisher's exact tests will be used to estimate differences for continuous and categorical variables. Model and parameter uncertainty will be tested using sensitivity analyses.

Ethics and dissemination – This will be the first study to examine the organisation of acute stroke care for IAT delivery on a national scale using discrete event simulation. There are no ethics or safety concerns regarding the dissemination of information, which includes publication in peer-reviewed journals and (inter)national conference presentations.

For peer review only

Strengths and limitations of the study

- The proposed simulation modelling study collects patient level data from all interventions centers in the Netherlands that provide IAT for acute ischaemic stroke patients.
- Information from prehospital stroke services including emergency medical services are included in the model.
- Information on costs associated with pathway set-up and innovations foreseen in acute stroke treatment are included.
- Model results are estimations which have to be tested in clinical practice.
- Input parameters for model building contain estimations of time delays and diagnostic procedures that may have changed over time.

For peer review only

INTRODUCTION

For acute ischaemic stroke patients, reperfusion therapies comprise intravenous thrombolysis (IVT) administered up to 4.5 hours after onset, and intra-arterial thrombectomy (IAT) up to 6 hours and in selected patients even up to 24 hours.[1-7] Functional recovery following treatment is strongly determined by the time interval between symptom onset and reperfusion. For every 9-minute delay along the care pathway, 1 in every 100 patients treated with IAT has a worse disability outcome (higher modified Rankin Scale score by 1 or more levels).[8] As such, timely arrival at the hospital and minimising the overall onset-to-treatment time is of critical importance. Following symptom onset patients are typically transported to the nearest hospital capable of administering IVT, which can be either a Primary Stroke Centre (PSC) or a Comprehensive Stroke Centre (CSC). In parallel with administering the bolus for IVT additional neuroimaging is performed in order to detect large vessel occlusion (LVO). In case of a LVO patients may become eligible for IAT after which transfer to a CSC is arranged, if patients are initially admitted to a PSC.

The transfer of patients to a CSC following IVT diagnostic work-up and/or treatment at a PSC is called the drip-and-ship (DS) approach. In addition to the DS approach patients may be transported directly to the CSC bypassing the PSC, which is called the Mothership (MS) approach. Currently there are no formal policies supporting the latter, as it necessitates accurate prehospital triage objectifying LVO with sufficient accuracy. Timely patient arrival and treatment with reperfusion therapies depends on a number of factors such as the geographical distribution and distances between PSCs and CSCs in relation to the location of the patient. Arrangements made by local emergency medical services concerning referral patterns of suspected stroke victims, and the quality and set-up of road networks also play a role, as does potential traffic congestion.

In the Netherlands sixteen medical centres that participated in the MR CLEAN trial are currently reimbursed for IAT. However, these hospitals are unevenly distributed leaving certain regions potentially underserved while in some areas there is a surplus of IAT centres. Insight into factors determining the potential optimal and most efficient way to organise acute stroke care is currently lacking but urgently needed to support clinicians and decision-makers. A key question is: what would be the optimal distribution of CSCs within a region, taking into account the effects of additional travel distances, available clinical expertise, treatment volumes and capacity of hospitals? Are there regions under- or overserved with CSCs and what would be the potential consequences of adding/removing CSCs within specific regions?

In this study, we aim to quantify pathway logistics, i.e. stroke onset and time delays associated with pre-hospital and intra-hospital care services among patients treated with IAT with or without IVT. From this baseline assessment a simulation model will be built that describes all activities and treatments performed along the acute stroke pathway. A generic model will be developed based on the set-up of IAT delivery in the region of Northern Netherlands, and extended using results of the MR CLEAN registry[9] and data of 5 randomised clinical trials (RCTs) performed in the 'Collaboration for new treatments in acute stroke (CONTRAST)' consortium (www.contrast-consortium.nl). The MR CLEAN registry is an ongoing, prospective, observational study in all centres that perform IAT in the Netherlands. Within CONTRAST 5 large clinical trials will be performed to test novel treatment strategies for stroke. This will be complemented by a pre-clinical program, development of a large biobank and the identification of challenges and solutions for organisational models. Retrospective data from the MR CLEAN registry combined with data from emergency medical services will serve as input for the development of a baseline simulation model for regional IAT delivery. Next, prospective data capturing using results

For peer review only - http://bmjopen.bmj.com/site/about/guidelines.xhtml

from ongoing trials performed within CONTRAST will be used to further develop the model. Simulation experiments will be performed to assess effectiveness and efficiency of alternative network topologies, i.e. primary and comprehensive stroke services (DS approach) versus a service delivery based on comprehensive and centralised care (MS approach). Model parameters will be extended by assigning unit costs to activities allowing for economic analyses. The latter will involve collection of detailed information on resource use allowing for micro-costing studies and subsequent use in cost-benefit and/or cost-effectiveness analyses.

Aims and hypotheses

Primary aim: to develop a simulation model for IAT in the Netherlands and to extend the model to reflect regional differences.

Secondary aims: to estimate the effectiveness of alternative network topologies on IVT and/or IAT treatment rates, geographical access to CSCs, time to treatment, treatment volumes and patient disability as measured by the modified Rankin Scale (mRS). Also, potential areas for pathway improvements in regional stroke care systems will be identified.

Tertiary aims: to extend the model with costs estimations of procedures and activities performed along the acute stroke pathway allowing for elaborate economic evaluation. Also, the assessment and potential implementation of new technologies such as the mobile stroke unit is foreseen through modelling.

METHODS

Study design

This study uses discrete event simulation modelling in which care pathways can be represented '*in silico*' by quantifying pathway performance.[10-12] In case of acute stroke treatment, information on time delays sustained by patients and diagnostic steps performed up to treatment with IVT and IAT are collected. In addition, patient demographics (age), exclusion and inclusion criteria and follow-up data up to three months post stroke will be included. Previous research has demonstrated that simulation models for representing the acute stroke pathway can be accurately developed in different settings.[13-17] Typically, simulation modelling involves a number of consecutive steps including model building, validation and experimentation. Model building starts with the conceptual representation of care pathways in building block such as incidents, time delays, queues, resources and ultimately outcome. Next the model needs to be populated through collection of empirical data reflecting actual pathway performance, for which a combination of real-world datasets are used. For hospital items we will rely on case report forms (CRFs) collected in the RCTs in the CONTRAST consortium involving sixteen hospitals in the Netherlands that provide IAT. In addition, collaboration with regional ambulance services will be set up in order to collect prehospital items such as time of 911 call, time spent on scene, transportation time to the hospital and general referral patterns. Real-world data collected in each step of the pathway will be analysed to distinguish statistical distributions using the statistical software package ExpertFit.[18] Based on the statistical distributions identified hypothetical patients passing through the model will be assigned a certain time delay, and diagnostic accuracy for each step along the pathway. Model outcomes include the proportion of patients treated with IAT, time to treatment and functional outcome after 90 days. These results will then be validated

comparing them to observed time delays and outcomes observed in clinical practice. Following model validation, experiments will be performed in which scenarios of alternative set-ups of the organisational models will be evaluated. Descriptions of the acute stroke pathway in both the DS and MS organisational models in Northern Netherlands are presented in Figures 1 and 2.

Study population

For this study we will use data from acute stroke patients that received IAT with or without IVT. A baseline model will be developed using a subset of patient information collected in the ongoing cohort study MR CLEAN Registry. Following the initial model patients treated in other regions in the Netherlands will be included. In later stages of model development alternative treatment modalities may become apparent such as treating patient with IAT up to 24 hours or including patients that receive prehospital treatment in a mobile stroke unit. The acute stroke phase is defined as the time window in which acute reperfusion therapies are being used.This can be the case up to a maximum of 24 hours following symptom onset. In these cases the parameters underlying the model will be changed accordingly. Modelling the hypothetical introduction of a mobile stroke unit will involve changing prehospital stroke management. In such a scenario an expedited and optimised process of initial diagnostic assessment and triage is assumed.

Public and patient involvement

Patients and public were involved in the conception of the topics to be addressed in the CONTRAST consortium. Study results will be disseminated through newsletters, poster presentations, and publications in newspapers, lay journals, and publication in peer-reviewed journals.

Data to be collected

An overview of all data items collected for the simulation model is presented in a table as supplementary material (Table S1. Overview of data collection). Based on this data, time intervals reflecting pathway efficiencies will be calculated, such as the time from symptom onset to computed tomography (CT)/ computed tomography angiography (CTA) and door-to-CT/CTA times for patients arriving at a PSC and after secondary transfer to the CSC, if necessary.

Hospital data

Within CONTRAST 5 RCTs are performed that serve as input for the proposed simulation modelling studies: MR ASAP (NL60258.078.17), MR CLEAN LATE (NL58246.078.17), MR CLEAN MED (ISCRTN76741621), MR CLEAN NO IV (ISRCTN80619088). Each intervention centre uses a standardised, customised and web-based electronic CRF to document all steps in the care process. All data will be entered locally in web-based database (OpenClinica). A copy of the CRF documents used in the clinical trials can be found online (www.contrast-consortium.nl). All studies aim to collect a large set of items containing, among others, a description of the workflow including time of symptom onset or last seen

For peer review only

well, time of arrival of the first hospital and whether the patient was transferred from another hospital. Within the hospital, time to CT scan and advanced imaging (computed tomography angiography/ magnetic resonance angiography/digital subtraction angiography/perfusion imaging) are collected according to local procedures. Also start of IVT (bolus infusion), patient arrival in the angiosuite, groin puncture, device attempts, recanalisation and sheath withdrawal/end of procedure are collected. In addition anesthetic team presence and management procedures are recorded.

Prehospital data

For the collection of prehospital data collaboration with regional ambulance services is being set-up. Items will include time delays, diagnostics and geographic information on referral patterns. Time delays include the time of 911 call, arrival at the location of the patient, departure at the location of the patient and arrival at the hospital. Diagnostic items include mode of referral (general practitioner, 911 or other), initial working diagnosis set by 911 dispatch and ambulance personnel and the level of urgent transportation (i.e. blue lights or other). Geographical information includes the postal codes of the location of the patient, departure location of the ambulance vehicle and location of the PSC and/or CSC.

Baseline data

Prior to the CONTRAST study, all hospitals contributed to the MR CLEAN Registry, an online and ongoing database to monitor implementation and safety of IAT in the Netherlands.[9] For CONTRAST the variables are similar to those collected in the MR CLEAN Registry. In addition, prehospital information available at regional ambulance services will be collected.

The baseline discrete event simulation model will be developed using data from 296 patients that received IAT at the University Medical Centre Groningen between July 2014 and November 2017. For these patients intrahospital time delays and diagnostic steps were already collected as part of the ongoing MR CLEAN Registry and subsequently used for model development and validation. In addition, prehospital items as described above were collected in collaboration with regional ambulance services.

Outcome measures

Outcomes will be estimated by the simulation model using parameter distributions as observed in clinical practice using real-world data.

Primary outcomes include IAT treatment rates and clinical outcome according to the modified Rankin Scale (mRS).

Secondary outcomes include process times such as symptom onset to hospital arrival, door to needle and groin puncture, arrival angiosuite, recanalization, onset-to-treatment time of IVT and/or IAT and interhospital transportation times for those patients that were transferred to a CSC after initial diagnosis and/or treatment with IVT at a PSC. In addition, the time interval of referral from the PSC to CSC is included.

Tertiary outcomes include treatment volume, direct costs, indirect costs and societal costs. This will include fixed and variable costs of providing services related to IAT delivery.

For peer review only

Ethics and dissemination

Both the MR CLEAN and CONTRAST databases include patients for which informed consent had been obtained, which was extended for use in our simulation modeling study. All data used for model building has been completely anonymized, performed by local researchers under auspices of the principal investigators for the MR CLEAN Registry and CONTRAST studies. Accordingly, no additional approval from our local ethics committee is required, as this represents a simulation study using an anonymized dataset.

In order to link data collected of patients treated with IAT after study enrollment in the hospital to corresponding data collected by ambulance services, identifiable information from patients will be used under a strict protocol compliant with the Dutch Personal Data Protection Act. This protocol for using prehospital ambulance data meets the criteria for a waiver of consent from the University Medical Centre Groningen Institutional Review Board, for which formal approval was already obtained. Following successful linking of pre-and inhospital data, note however, that also these data were anonymized. Prior to the exchange of information, a data transfer and processing agreement was agreed upon and signed by each provider of regional ambulance services.

Study results will be published in peer-reviewed journals and (inter)national conference presentations. Centres that have participated in the study will have access to their own data and results.

Study sites

Sixteen hospitals providing IAT will participate. In addition, regional ambulance services in the catchment area of intervention centres will be asked to participate. The overview of intervention centres, their catchment area and regional ambulance services active in the region are provided as supplementary material (Figure S1. Overview of IAT centres and ambulance services).

Statistical analyses

Descriptive statistics will be used to characterise the population and regional differences. Mann-Whitney U and Fisher's exact tests are used to estimate the differences for continuous and categorical variables. Model and parameter uncertainty will be tested using sensitivity analyses.

Approach to missing data

Multiple imputation techniques will be used to handle missing data. The impact of missing data will be analysed by performing sensitivity analyses. Once missing data or errors are identified, all corrections made will be documented. All data will be documented and displayed in an appropriate flow diagram.

Model validation

Internal validation will be performed by comparing model performance with observed performance of the stroke system to ascertain whether the model represented the real system

For peer review only

accurately. External validation of the baseline model will be performed by using the pathway performance of other PSCs and CSCs participating in the CONTRAST consortium. As such, the model developed for one region will be extended to other parts of the Netherlands by changing the distributions underlying the simulation model. This means that the model is re-populated by real-world data originating from different regions, including both intrahospital and prehospital data. As we will adopt one uniform method for data collected in both the pre- and intrahospital pathway, similar procedures for model building, validation and experimentation can be followed.

Study organisation

The CONTRAST consortium is a nationwide collaboration of clinical and translational scientists from all academic and large clinical centres who want to act together to improve the treatment of acute stroke in the Netherlands. Five large acute stroke trials to test novel treatment strategies will be performed including: prehospital augmentation of collateral blood flow and blood pressure management (MR ASAP), antithrombotics to prevent microvascular occlusion after IAT (MR CLEAN MED), immediate IAT without preceding thrombolysis (MR CLEAN NO IV),IAT in the 6 to 24 hour time window (MR CLEAN LATE) and minimally invasive endoscopy guided surgery for intracerebral hemorrhage (Dutch ICH trial). Discrete event simulation modelling is applied with data from these trials to optimise acute stroke care delivery.

DISCUSSION

This simulation modelling study will evaluate the set-up of acute stroke services for patients receiving IAT in a specific geographical setting, i.e., the Netherlands. However, the model foreseen may also serve as a planning and evaluation tool for other regions and countries. Already from an early phase onwards the potential consequences of the clinical trials performed in CONTRAST on the organisation of acute stroke care will be assessed. Benefits of innovations foreseen in prehospital as well as hospital based diagnosis and treatment will be estimated on a regional and national scale. Likewise, the optimal organisation ensuing from these re-organising care processes may be identified and taken into account given available resources and capacity. Another important aspect that will be studied is how re-distribution of PSCs and CSCs within certain regions will affect time to treatment for both IVT and IAT.[19] Experiments from simulation models will help to elucidate factors determining optimal stroke pathway set-up and identify potential targets for further optimisation of pathway performance. Simulation modelling also allows for making exact estimations on effects on patients health and costs when adding or removing IAT centres in specific regions, thereby supporting clinicians and policy makers in decision making.[15]

Our simulation modelling approach has several strengths. First, the prospective design and uniform methods for data collection both in the pre- and intrahospital phase will ensure a high quality of data capture. The use of real-world data will support the external validation of results obtained and is a unique feature of the proposed modelling study. Also in terms of efficiency simulation modelling is an attractive option, as it utilizes already collected information in clinical trials and by ambulance services. Second, the model will have a comprehensive scope, contrary to current improvement approaches characterised by mono-dimensional interventions.[20-22] Implementation problems such as how many CSCs would a region need or where they should ideally be located require a multi-faceted solution, and

could therefore benefit from a modelling approach. Data used for model input will be collected in various regions of the Netherlands reflecting differences in access to PSCs and CSCs, road networks and potential traffic congestion. Third, the model builds on a previously developed simulation model for IVT treatment in Northern Netherlands.[10] As such we have demonstrated that model building and validation is feasible. Finally, the model can be extended to include unit costs for elaborate economic analyses. The comprehensive character of the modelling approach also allows considering potential interaction effects between variables, and has a quadruple dimensional approach in terms of measuring outcomes, as it can capture effects on clinical outcomes, time to treatment, referral patterns and costs of processes associated with IVT and IAT delivery.

Serving as a precursor for clinical validation, results obtained from modelling studies should always be interpreted with care as these results do not represent actual patients. However, from an economics and business approach each treatment can be represented as the sum of time delays and diagnostics performed along the care pathway, and as such can be quantified and in turn represented '*in silico*' by computer models. One of the main questions is whether there is benefit in bypassing the nearest PSC to transport the patient directly to a CSC. At the moment there is no consensus which prehospital scale to use in order to quantify stroke severity, and no agreed upon triage instrument exists to guide transfer of patients to specific hospitals. Typically, in case of a positive trial results in CONTRAST this will have a direct impact on the organisational model for IAT delivery, either by suggesting to bypass PSCs (MR CLEAN NO IV), by extending the time window for reperfusion therapies (MR CLEAN LATE) or to change prehospital stroke management (MR ASAP). As a consequence alternative set-up of services is foreseen, in which accurate estimations on effectiveness, time to treatment and cost-effectiveness are required to support decision-making.

In summary, the proposed modelling study will investigate current organisational models for IAT delivery in the Netherlands. Based on input from real-world clinical studies performed in the CONTRAST consortium insight into current treatment delays and workflow performance of IAT centres will be obtained. Next, simulation modelling studies will support design of optimal distribution of IAT centres, in order to improve patient outcomes and increase the proportion of patients eligible for IAT.

Acknowledgements We acknowledge the support of the Netherlands Cardiovascular Research Initiative which is supported by the Dutch Heart Foundation (CVON2015-01: CONTRAST), the support of the Brain Foundation Netherlands (HA2015.01.06), and the support of Health~Holland, Top Sector Life Sciences & Health (LSHM17016) and of Medtronic.

Contributors ML, WM, DJZ, MU and EB designed the study with MU and EB as principal investigators. ML, MU and EB applied for, received and organised study funding. ML drafted the manuscript, WM, DJZ, MU and EB critically revised the manuscript for intellectual content and approved the final version of the manuscript for publication.

Funding The CONTRAST consortium is supported by Netherlands Cardiovascular Research Initiative, an initiative of the Dutch Heart Foundation, by the Brain Foundation Netherlands

and powered by Health~Holland, Top Sector Life Sciences and receives unrestricted funding from Medtronic.

Competing interest statement The authors declare that there is no conflict of interest.

Figure 1. Description of the drip-and-ship model.

Figure 2. Description of the mothership model.

References

1 . Tissue plasminogen activator for acute ischemic stroke. The National Institute of Neurological Disorders and Stroke rt-PA Stroke Study Group. *N Engl J Med* 1995;333:1581-7 doi:10.1056/NEJM199512143332401.

2 Berkhemer OA, Fransen PS, Beumer D, et al. A randomized trial of intraarterial treatment for acute ischemic stroke. *N Engl J Med* 2015;372:11-20 doi:10.1056/NEJMoa1411587 [doi].

3 Campbell BC, Mitchell PJ, Kleinig TJ, et al. Endovascular therapy for ischemic stroke with perfusion-imaging selection. *N Engl J Med* 2015;372:1009-18 doi:10.1056/NEJMoa1414792 [doi].

4 Goyal M, Demchuk AM, Menon BK, et al. Randomized assessment of rapid endovascular treatment of ischemic stroke. *N Engl J Med* 2015;372:1019-30 doi:10.1056/NEJMoa1414905 [doi].

5 Jovin TG, Chamorro A, Cobo E, et al. Thrombectomy within 8 hours after symptom onset in ischemic stroke. *N Engl J Med* 2015;372:2296-306 doi:10.1056/NEJMoa1503780 [doi].

6 Saver JL, Goyal M, Bonafe A, et al. Stent-retriever thrombectomy after intravenous t-PA vs. t-PA alone in stroke. *N Engl J Med* 2015;372:2285-95 doi:10.1056/NEJMoa1415061 [doi].

7 Nogueira RG, Jadhav AP, Haussen DC, et al. Thrombectomy 6 to 24 Hours after Stroke with a Mismatch between Deficit and Infarct. *N Engl J Med* 2018;378:11-21 doi:10.1056/NEJMoa1706442 [doi].

8 Saver JL, Goyal M, van der Lugt A, et al. Time to Treatment With Endovascular Thrombectomy and Outcomes From Ischemic Stroke: A Meta-analysis. *JAMA* 2016;316:1279-88 doi:10.1001/jama.2016.13647 [doi].

9 Jansen IGH, Mulder MJHL, Goldhoorn RB, et al. Endovascular treatment for acute ischaemic stroke in routine clinical practice: prospective, observational cohort study (MR CLEAN Registry). *BMJ* 2018;360:k949 doi:10.1136/bmj.k949 [doi].

For peer review only

10 Lahr MM, van der Zee DJ, Vroomen PC, et al. Thrombolysis in acute ischemic stroke: a simulation study to improve pre- and in-hospital delays in community hospitals. *PLoS One* 2013;8:e79049 doi:10.1371/journal.pone.0079049 [doi].

11 Ramwadhdoebe S, Van Merode GG, Boere-Boonekamp MM, et al. Implementation by simulation; strategies for ultrasound screening for hip dysplasia in the Netherlands. *BMC Health Serv Res* 2010;10:75 doi:10.1186/1472-6963-10-75.

12 Heeg BM, Damen J, Buskens E, et al. Modelling approaches: the case of schizophrenia. *Pharmacoeconomics* 2008;26:633-48 doi:2682 [pii].

13 Holodinsky JK, Williamson TS, Demchuk AM, et al. Modeling Stroke Patient Transport for All Patients With Suspected Large-Vessel Occlusion. *JAMA Neurol* 2018;75:1477-86 doi:10.1001/jamaneurol.2018.2424 [doi].

14 Phan TG, Beare R, Chen J, et al. Googling Service Boundaries for Endovascular Clot Retrieval Hub Hospitals in a Metropolitan Setting: Proof-of-Concept Study. *Stroke* 2017;48:1353-61 doi:10.1161/STROKEAHA.116.015323 [doi].

15 Lahr MM, van der Zee DJ, Luijckx GJ, et al. Centralising and optimising decentralised stroke care systems: a simulation study on short-term costs and effects. *BMC Med Res Methodol* 2017;17:5,016-0275-3 doi:10.1186/s12874-016-0275-3 [doi].

16 Monks T, Pitt M, Stein K, et al. Maximizing the Population Benefit From Thrombolysis in Acute Ischemic Stroke: A Modeling Study of In-Hospital Delays. *Stroke* 2012;43(10):2706-11 doi:10.1161/STROKEAHA.112.663187.

17 Churilov L, Fridriksdottir A, Keshtkaran M, et al. Decision support in pre-hospital stroke care operations: a case of simulation to improve eligibility of acute stroke patients for thrombolysis treatment. Computer & Operations Research, Available online 14 July 2012.

18 Law AM. ExpertFit Version 8 User's Guide. Tuscon, Arizona: Averill M. Law & Associates 2011.

19 Allen M, Pearn K, James M, et al. Maximising access to thrombectomy services for stroke in England: A modelling study. *Eur Stroke J* 2019;4:39-49 doi:10.1177/2396987318785421 [doi].

20 Jeon SB, Ryoo SM, Lee DH, et al. Multidisciplinary Approach to Decrease In-Hospital Delay for Stroke Thrombolysis. *J Stroke* 2017;19:196-204 doi:10.5853/jos.2016.01802 [doi].

21 Psychogios MN, Behme D, Schregel K, et al. One-Stop Management of Acute Stroke Patients: Minimizing Door-to-Reperfusion Times. *Stroke* 2017;48:3152-5 doi:10.1161/STROKEAHA.117.018077 [doi].

22 Schregel K, Behme D, Tsogkas I, et al. Effects of Workflow Optimization in Endovascularly Treated Stroke Patients - A Pre-Post Effectiveness Study. *PLoS One* 2016;11:e0169192 doi:10.1371/journal.pone.0169192 [doi].

Figure legends

Figure 1. Description of the drip-and-ship model.

Figure 2. Description of the Mothership model.

For peer review only

1

For peer review only

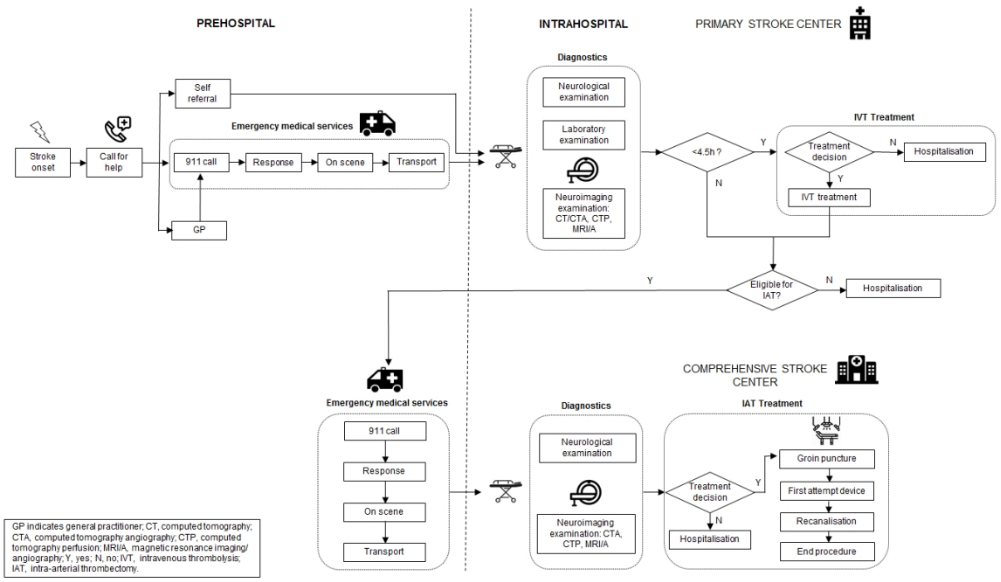

Figure 1

356x206mm (96 x 96 DPI)

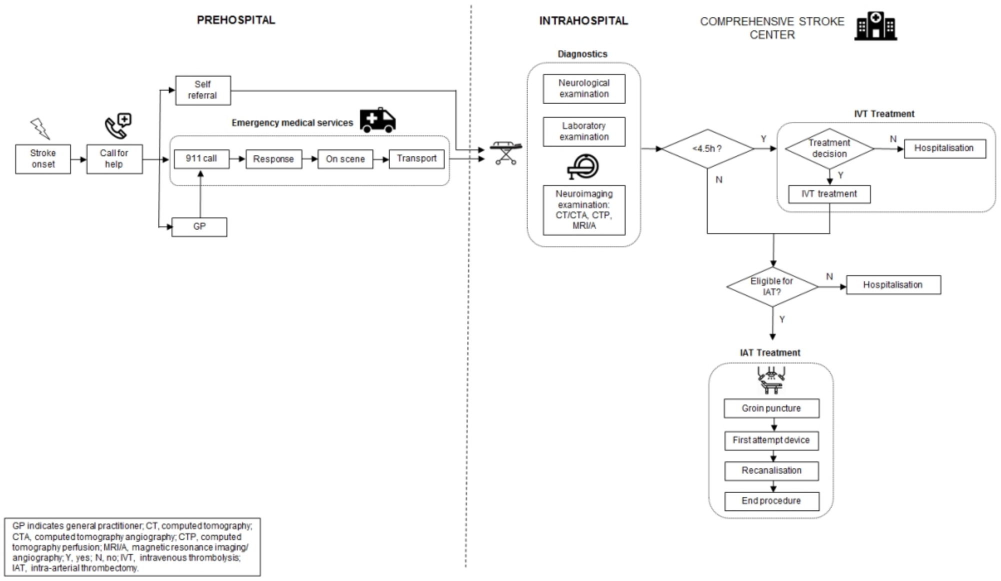

Figure 2

356x207mm (96 x 96 DPI)

Supplementary files

Table S1. Overview of data collection [Table S1.docx] – supplementary material for “Rationale and design of a simulation modelling approach for intra-arterial thrombectomy in the Netherlands” by Lahr M.M. et al.

Table 1. Overview of data collection**.**

**Item**	**Description**	**Source**
Prehospital items		
Symptom onset	Exact time of stroke onset, last seen well or symptoms noticed	CRF
Mode of referral	Initial call for help directed at either the GP, 911 emergency services or via self-transport	Ambulance database
Time first 911 call	Moment of first 911 call as documented at the control centre of the regional ambulance service	Ambulance database
Time second 911 call†	Moment of second 911 call as documented at the control centre of the regional ambulance service	Ambulance database
Time departure ambulance patient	Moment of ambulance departure in the direction of the patient	Ambulance database
Time departure ambulance hospital	Moment of ambulance departure in the direction of the PSC for transfer to the CSC	Ambulance database
Time ambulance arrival patient	Moment of ambulance arrival at the location of the patient	Ambulance database
Time ambulance arrival first hospital	Moment of ambulance arrival at the first hospital	Ambulance database
Time ambulance arrival second hospital†	Moment of ambulance arrival at the second hospital	Ambulance database
Time ambulance arrival PSC†	Moment of ambulance arrival at the PSC to transfer a patient to the CSC	Ambulance database
Procedures at location patient	All procedures performance by ambulance personnel at the location of the patient	Ambulance database
Postal code patient	Exact geographic location of the patient	Ambulance database
Postal code hospital	Exact geographical location of the destination hospital	Ambulance database
Intrahospital items		
Time of arrival ER first hospital	Moment of patient entry into the hospital electronic system	CRF
Time of arrival ER intervention hospital	Moment of patient entry into the hospital electronic system	CRF
Time of CT scan first hospital	Moment of CT scan performance in the first hospital	CRF
Time of CT scan second hospital	Moment of CT scan performance in the second hospital	CRF
Time of CTA scan first hospital	Moment of CTA scan performance in the first hospital	CRF
Time of CTA scan second hospital	Moment of CTA scan performance in the second hospital	CRF

For peer review only

Time of IV alteplase bolus (if given)	Moment of intravenous thrombolysis	CRF
Time of angiosuite arrival	Moment of patient arrival at the angiosuite	CRF
Time groin puncture	Moment of groin puncture	CRF
Time device attempt	Moment of device attempt	CRF
Time recanalisation	Time of recanalisation	CRF
Time of end procedure	Moment of sheath withdrawal/end of procedure	CRF

CRF indicates case report form; PSC, primary stroke centre; CSC, comprehensive stroke centre; CT, computed tomography; CTA, computed tomography angiogram.

†In case of intravenous thrombolysis at the primary stroke centre followed by intra-arterial thrombectomy at the comprehensive stroke centre (drip-and-ship approach).

Figure S1. Overview of IAT centres and ambulance services [Supplementary figure S1.tiff] - supplementary material for "Rationale and design of a simulation modelling approach for intra-arterial thrombectomy in the Netherlands" by Lahr M.M. et al.

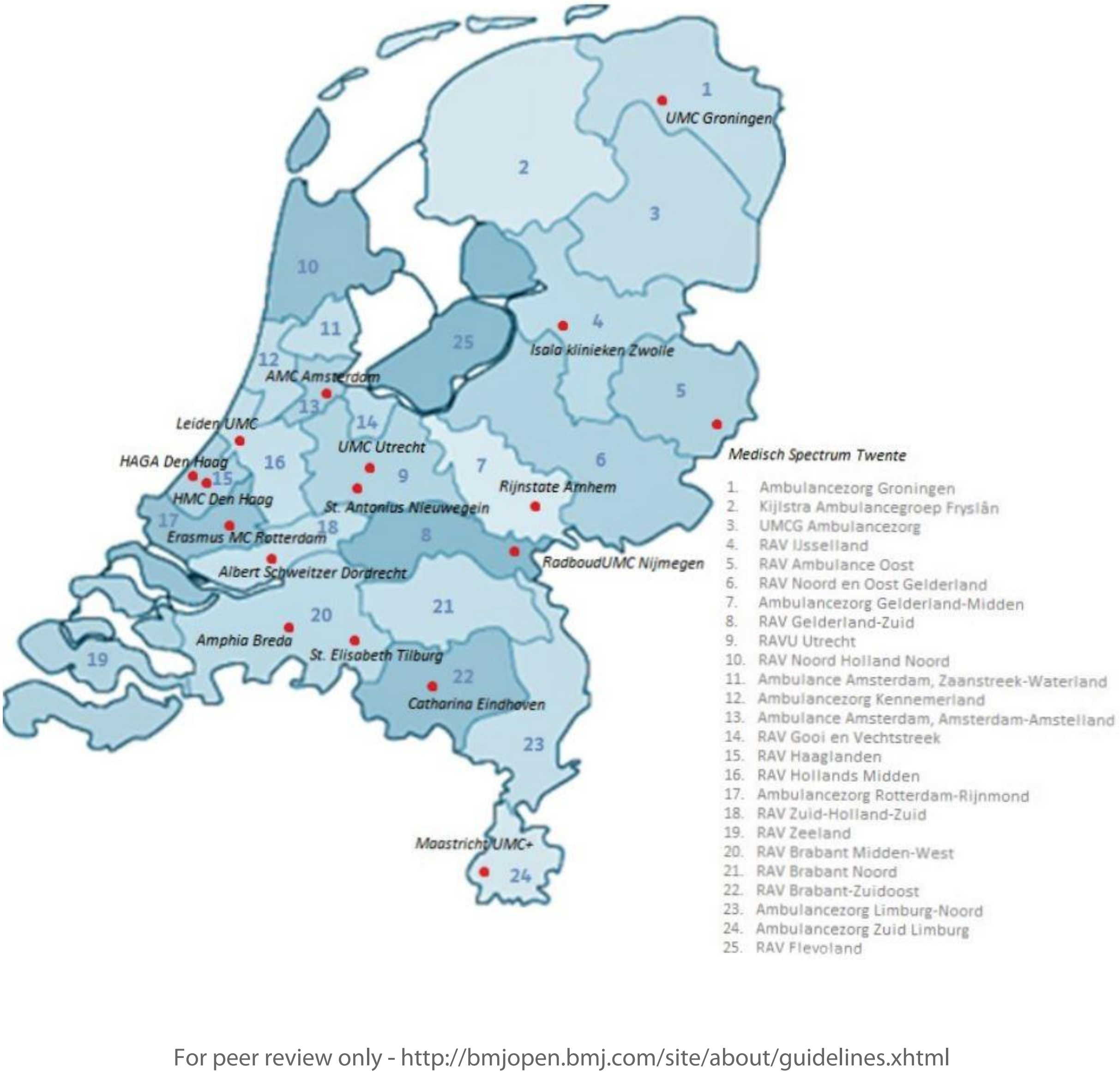

Supplement: Author's manuscript [file bmjopen-2019-032754.draft_revisions.pdf]
